# A Non-Controlled Study of Cognitive Behavior Therapy for Panic Disorder in Veterans

**DOI:** 10.3390/bs16081453

**Published:** 2026-08-21

**Authors:** Daniel F. Gros, Jeffrey M. Pavlacic

**Affiliations:** 1 Mental Health Service, Ralph H. Johnson Veterans Affairs Health Care System, Charleston, SC 29401, USA; 2 Department of Psychiatry and Behavioral Sciences, Medical University of South Carolina, Charleston, SC 29425, USA

**Keywords:** veterans, panic disorder, psychotherapy, comorbidity

## Abstract

Panic disorder is a highly prevalent and impairing diagnosis in veterans. Despite consistent efficacy findings in community samples, few studies have investigated evidence-based psychotherapies in veterans with panic disorder, limiting the understanding of their utility and deployment in Veterans Affairs (VA) settings. The present study preliminarily investigated cognitive behavioral therapy (CBT) for panic disorder via a non-controlled design in a treatment-seeking veteran sample with panic disorder at the VA. Of the 41 veterans initiating treatment, 28 (68.3%) participants completed at least eight sessions of psychotherapy. In general, participants evidenced medium-to-large post-treatment effects across disorder-specific symptoms (panic and anxiety symptomatology), comorbid presentations (symptoms of depression and posttraumatic stress disorder), and overall functioning. Disorder-specific symptom effects remained stable for six months after psychotherapy was terminated. In addition, the delivery of CBT for panic disorder was further supported by positive indices of treatment feasibility, including treatment engagement, homework completion and engagement, and high participant satisfaction ratings. While recognizing that this study did not include a control group, the findings provide limited support for CBT treatment of panic disorder in veterans’ settings, findings that will require confirmation in a multisite controlled clinical trial.

## 1. A Non-Controlled Study of Cognitive Behavior Therapy for Panic Disorder in Veterans

The *Diagnostic and Statistical Manual of Mental Disorders* (DSM-5; American Psychiatric Association, 2013) defines a panic attack as an abrupt surge of fear and physiological and cognitive symptoms. Panic disorder involves recurrent panic attacks as well as behavioral avoidance and fear of having a panic attack in the future. Prevalence rates of panic disorder are 2.7% for community samples (Kessler et al., 2006) and 6.8% in primary care settings (Gros et al., 2023; Kroenke et al., 2007). Panic disorder is related to physical health concerns, in addition to elevated healthcare utilization (Bystritsky et al., 2010; Gros et al., 2023; Hendriks et al., 2016; Horenstein & Heimberg, 2020; Rubin et al., 2000).

Cognitive behavioral therapy (CBT) for panic disorder has received the most empirical support (Otto & Deveney, 2005; Rayburn & Otto, 2003). The CBT model explains a panic attack as an intense anxiety response (e.g., physiological activation) to subjective rather than objective danger (e.g., car accident), with panic attacks characterized by strong negative cognitions, including, but not limited to, fears of embarrassment, loss of control, or death (Rayburn & Otto, 2003). Fears of future panic attacks sustain heightened vigilance of associated internal cues that trigger further misinterpretation, fears, and accelerated reactivity when internal cues are detected (e.g., natural heart racing from physical activity is misinterpreted as an approaching panic attack, causing increased anxiety, physiological reactivity, and eventual panic and avoidance). Avoidance of internal (e.g., ingesting caffeine—heart racing) and external (e.g., crowded location—fear of prevented escape) stimuli prevents future re-learning or re-establishing a sense of safety in feared situations. Effective CBT treatment approaches for panic disorder incorporate cognitive restructuring and exposure interventions to challenge fears of anxiety sensations and reduce avoidance of physical sensations and related situations that are perceived to be dangerous. Across meta-analyses, CBT for panic disorder has evidenced large effect sizes for primary panic symptoms as well as for comorbid symptomatology, with sustained improvements over time (Otto & Deveney, 2005; Rayburn & Otto, 2003; Roy-Byrne et al., 2006).

Despite the research in community healthcare settings, far less is known regarding panic disorder in veterans and military personnel (Barrera et al., 2013a, 2013b; Gros et al., 2011). This may be in part due to the prioritization of other mental health conditions, such as posttraumatic stress disorder (PTSD) and major depressive disorder (MDD), in terms of standard assessment batteries (including PHQ-9 and PTSD Checklist for DSM-5) and evidence-based psychotherapy (EBP) rollout efforts (e.g., including CBT for MDD, Acceptance and Commitment Therapy for MDD, Cognitive Processing Therapy for PTSD, and Prolonged Exposure for PTSD) via the Department of Veterans Affairs (VA). There are no standardized measures or EBPs widely disseminated for panic disorder in the VA. Despite a general lack of dissemination or prioritization, initial findings have demonstrated slightly higher prevalence rates (6.1 to 8.3%) of panic disorder in veterans across epidemiological studies compared to community samples (Barrera et al., 2013b; Gros et al., 2011). Symptoms of panic and agoraphobia in veterans have been associated with severe physical health and mental health impairment and limited social functioning (Barrera et al., 2013b; Gros et al., 2011), as well as with comorbid depressive symptoms and suicidal ideation (Gros et al., 2023).

To date, panic disorder interventions have been tested using pilot designs in veterans (Teng et al., 2008, 2015). Teng et al. (2008) investigated 18 veterans (12 completed treatment) with a primary diagnosis of PTSD and a secondary diagnosis of panic disorder via CBT of panic disorder, with promising treatment findings at post-treatment and 3-month follow-up compared to a psychoeducation condition. Teng et al. (2015) also investigated 44 veterans with panic disorder via an intense group psychotherapy (two 6 h groups across two days) and demonstrated promising findings for symptoms of panic disorder as well as for comorbid symptoms of PTSD. However, through these preliminary studies, findings for traditional CBT for panic disorder (versus intensive group therapy) in veterans with panic disorder (versus veterans with PTSD and panic disorder comorbidity) are unavailable. More specifically, no studies have investigated how veterans seeking treatment specifically for panic disorder symptoms respond to traditional CBT for panic disorder.

Differences in mental health symptoms, diagnoses, and treatment engagement have been well documented between veteran and civilian samples, with generally poorer health and health behaviors found in veterans (Hoerster et al., 2012; Oster et al., 2017; Williamson et al., 2023). In terms of EBP outcomes, researchers have hypothesized that veterans may evidence poorer outcomes due to the extended, repeated, and intense nature of deployment-related traumatic events, as well as higher rates of diagnostic comorbidity compared to civilians (Steenkamp et al., 2015). Given these findings, it is essential that ‘established treatments’, such as CBT for panic disorder, are also investigated in veteran samples to better understand their treatment effects across presentations. The present study investigated CBT for panic disorder in treatment-seeking veterans with panic disorder at a large VA Health Care System (VAHCS). The study evaluated primary outcomes (panic and anxiety symptoms) and secondary, comorbid symptoms (PTSD and depression), as well as overall impairment immediately after psychotherapy and at 6-month follow-up. Based on the findings in civilian samples (Roy-Byrne et al., 2006) and related preliminary therapy findings in veterans (Teng et al., 2008, 2015), we hypothesized that veterans with panic disorder would evidence significant symptom reductions in primary symptoms of panic disorder during treatment and that outcomes would be maintained at the 6-month follow-up.

## 2. Materials and Methods

### 2.1. Participants

Forty-one veterans with panic symptoms were recruited from outpatient mental health services at a large southeastern VAHCS. Inclusion criteria were as follows: (1) principal diagnosis of panic disorder adhering to criteria from the DSM-5 as assessed using the Anxiety Disorders Interview Schedule 5 (ADIS-5) (Brown, 2014); and (2) 18–80 years of age. Exclusion criteria were: (1) psychiatric hospitalization or a suicide attempt within the past 60 days; (2) beginning a new psychotropic medication within the past 30 days; and/or (3) reporting a severe acute or chronic medical condition likely to interfere with study participation/procedures (e.g., chronic obstructive pulmonary disease preventing participation in exposure practices). After study initiation, psychiatric medication adjustments were permitted and monitored.

### 2.2. Study Procedures

The VAHCS Research and Development and affiliate Institutional Review Board committees approved the study procedures. Recruitment took place between November 2020–June 2024. Therapy-seeking VAHCS patients with symptoms of panic attacks were referred to the study by their primary care or mental health providers. Upon study referral, patients identified as eligible completed a comprehensive intake assessment. The intake assessment included informed consent, a diagnostic interview (ADIS-5), and self-report measures of different psychiatric and functioning outcomes. Due to the overlapping COVID pandemic, all procedures were completed via VAHCS video telehealth with paper documentation exchanged via the United States Postal Service from November 2020 to September 2023, at which point procedures were transitioned to electronic delivery and in-person appointments (when requested) for the remainder of the study. If meeting study inclusion and exclusion criteria, participants were assigned to a study therapist to complete weekly sessions of CBT for panic disorder (Craske & Barlow, 2022). Study measures were repeated at immediate post-treatment and at six-month follow-up (six months after completion of treatment). Participants were compensated up to USD 300 for their completion of the study measures.

### 2.3. Cognitive Behavioral Therapy for Panic Disorder

CBT for panic disorder is designed to modify anxious thoughts and challenge situational and interoceptive avoidance to aid in resolving the symptoms of panic disorder (Craske & Barlow, 2022). The study protocol involved up to twelve 45–60 min weekly sessions, although participants could elect to finish after Session 8, based on participant–therapist agreement on sufficient symptom improvement. The treatment is separated into three core treatment components: (1) psychoeducation on anxiety and panic from a cognitive-behavioral perspective (e.g., nature of anxiety, three components of emotions, and CBT model applied to panic disorder); (2) cognitive interventions (e.g., identifying anxious thoughts, labelling cognitive distortions, and countering anxious thoughts); and (3) behavioral interventions (e.g., situational exposures to feared or avoided situations and interoceptive exposures to feared somatic sensations). All participants were guided through these treatment components via a session-by-session protocol, and with weekly homework assignments based on session topics (e.g., education materials for psychoeducation, thought records for cognitive interventions, and exposure practices for behavioral interventions). As reviewed previously, there is extensive evidence supporting the efficacy of CBT for panic disorder (Porter & Chambless, 2015; Rayburn & Otto, 2003).

The therapy was delivered by master’s-level therapists either face-to-face or via video telehealth, based on participant preferences. All therapists received extensive training in the protocol via attending a training workshop, completing practice cases with supervision, and receiving ongoing supervision throughout the duration of the study. Acceptable treatment fidelity was confirmed via a review of 20% of treatment session recordings on a session-specific 5-point rating scale (*M* = 4.4; *SD* = 0.7).

### 2.4. Measures

#### 2.4.1. Anxiety Disorder Interview Schedule 5 (ADIS-5)

The ADIS-5 assesses current diagnostic criteria and severity scores across DSM-5 diagnoses (Brown, 2014). The ADIS-5 has excellent psychometric properties (Brown, 2014). Initial screenings for panic attacks are followed by assessment of the relevant physical sensations (e.g., sweating, chills, numbing) and cognitive symptoms (e.g., fear of losing control) of a panic attack on a nine-point severity scale. Additional DSM criteria are assessed (e.g., worried or concerned about having another panic attack), in addition to a severity rating across significant areas of avoidance. Finally, participants receive overall ratings for interference and distress associated with their diagnosis. In the present study, 20% of interviews were scored by an independent rater, with acceptable inter-rater agreement for panic disorder (92%).

#### 2.4.2. Illness Intrusiveness Ratings Scale (IIRS)

The IIRS (Devins, 2010) includes 13 items and measures the extent to which psychiatric symptomatology interferes with important life domains. The IIRS has been shown to have strong psychometric properties (Devins, 2010). The IIRS demonstrated excellent internal consistency at baseline (α = 0.91). The IIRS was used to measure functional impairment, with higher scores reflecting more elevated functional impairment.

#### 2.4.3. Panic Disorder Severity Scale (PDSS)

The PDSS includes seven items and measures the frequency and distress of panic attacks and related symptoms “during the past week.” Items are scored on a 0–4 scale (Shear et al., 1997); higher scores reflect more panic symptoms. The scale has excellent psychometric properties (Shear et al., 1997). The PDSS showed good internal consistency at baseline (α = 0.84).

#### 2.4.4. PHQ-9

The PHQ-9 includes nine items and measures depressive symptoms “over the last 2 weeks.” Items are scored on a 0–3 scale (“Not at all” to “Nearly every day”) (Kroenke et al., 2001); higher scores suggest more severe depressive symptoms. The PHQ-9 has good psychometrics (Kroenke et al., 2001) and showed good internal consistency at baseline (α = 0.89).

#### 2.4.5. Post-Treatment Therapist Review

The Post-Treatment Therapist Review (PTTR) questionnaire was completed at posttreatment by the treating therapist (Shapiro & Gros, 2021). The PTTR inquired about a variety of post-treatment outcomes (e.g., session completion, homework adherence, and engagement). The present study utilized PTTR Item 1 (“total sessions attended”) and PTTR Item 4 (“total number of weeks in treatment”). In addition, PTTR Item 7 (“Participants understanding of the treatment model”), PTTR Item 8 (“weekly average percentage of homework completed”), and PTTR Item 10 (“weekly average homework engagement) were included and scored from 0 (none) to 6 (maximum). The PTTR Item 9 (“weekly average homework engagement”) was also used and rated from 0% to 100%.

#### 2.4.6. PTSD Checklist for DSM-5 (PCL-5)

The PCL-5 includes 20 items measuring DSM-5 PTSD symptoms experienced (Bovin et al., 2016). Items are scored on a 0–4 scale; higher scores suggest more severe PTSD symptoms. The PCL has excellent psychometric properties (Bovin et al., 2016). The PCL-5 showed excellent internal consistency at baseline (α = 0.95).

#### 2.4.7. Satisfaction with Therapy and Therapist Scale—Revised (STTS-R)

The STTS-R is a 12-item questionnaire that assesses a patient’s satisfaction with the therapy (seven items) provided, as well as satisfaction with their therapist (five items) (Oei & Green, 2008). The STTS-R was only assessed in treatment completers (immediate post-treatment assessment). Items are scored on a 1–5 scale (“strongly disagree” to “strongly agree”) with higher scores consistent with greater satisfaction. The STTS-R has demonstrated excellent psychometric properties across studies and demonstrated acceptable internal consistency in the present study (α = 0.92).

#### 2.4.8. State-Trait Inventory for Cognitive and Somatic Anxiety—Trait Version (STICSA)

The STICSA includes 21 items and measures trait cognitive and somatic anxiety (Gros et al., 2007; Ree et al., 2008), with higher scores indicative of more severe anxiety. Both the cognitive and somatic subscales are supported by factor analyses, with each subscale suggesting good internal consistency reliability (Gros et al., 2007). The STICSA shows stability across stress manipulations (Ree et al., 2008). In calculating an overall internal consistency coefficient at baseline, the STICSA demonstrate excellent internal consistency (α = 0.94).

### 2.5. Data-Analytic Plan

Linear mixed-effects models (LMEM) with restricted maximum likelihood were used to examine changes in various symptom and functional outcomes using baseline data, post-treatment data, and follow-up data. We used LMEMs to maximize baseline, post-treatment, and follow-up data, considering missing data and the relatively small sample size used in the present study. LMEMs were calculated in JASP. First, we entered time as a fixed effect (baseline, post-treatment, follow-up), with the participant included as a random effect to account for the nested nature of the data. We used the Satterthwaite approximation to determine *df* across models, delineated below for each model. Separate LMEMs were calculated for panic (PDSS), PTSD (PCL-5), depression (PHQ-9), cognitive anxiety (STICSA), somatic anxiety (STICSA), and functional impairment (IIRS). An exploratory linear regression was tested to see if PHQ-9 and PCL-5 scores predicted changes in panic symptoms (PDSS). For the LMEMs, effect sizes were calculated using Feingold’s method (e.g., Feingold, 2009); effect sizes are presented for baseline/post-treatment and baseline/follow-up. Contrasts comparing marginal means used a Holm *p*-value adjustment.

Of note, a higher rate of missing data was experienced in the present study related to procedural changes required to adjust to the COVID pandemic (e.g., assessments were completed via video telehealth appointments; questionnaire packets were exchanged via United States Postal Service). Missingness varies intermittently across outcomes from the original 41 participants. We have added the number of individuals with at least one data point across the outcomes examined, ranging from 36–38 participants. Missing data informed the decision to use an LMEM, as these model estimates still consider participants with less than three data points. We intended to analyze data for all individuals who were randomized and provided baseline data. Given data missingness, a modified intent-to-treat approach was used. LMEMs assume that data are missing at random.

## 3. Results

### 3.1. Participant Characteristics

**Figure 1:**
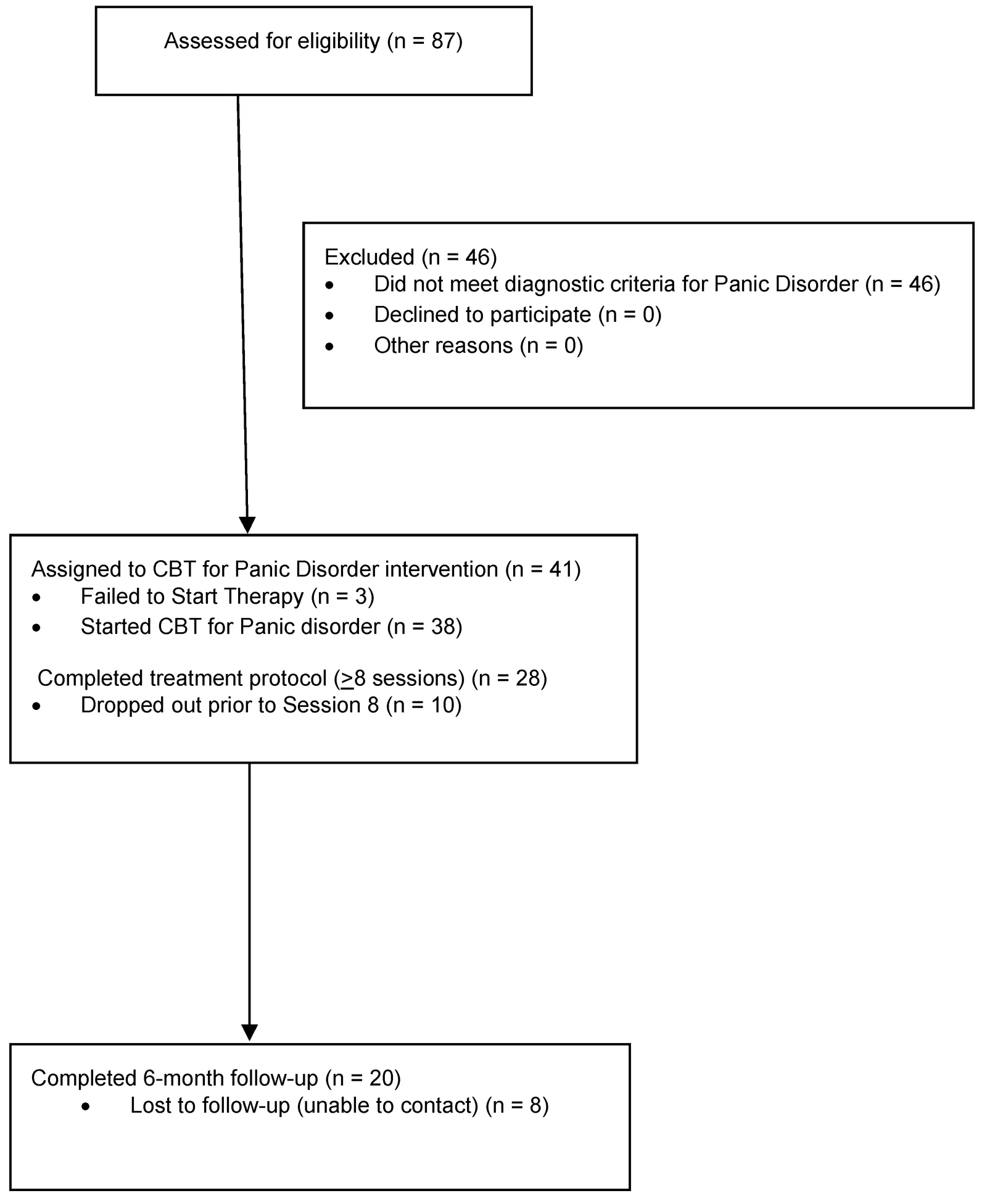
Consolidated standards of reporting trials (CONSORT) flow diagram showing participants’ flow.

Participants were 43.2 years old on average (*SD* = 12.8). Participants were predominately men (73.2%) and identified as either White (58.5%) or Black (39%). Half of the sample reported that they were unemployed, with 70.7% endorsing a VA service-connected disability. Roughly half of the sample reported serving in the Army (48.5%), with one-third reporting deployment to Operations Iraqi/Enduring Freedom (33.3%), and with 42.4% reporting combat exposure overall. From a diagnostic perspective, most participants endorsed diagnostic comorbidity (82.9%) with MDD (61.0%) and PTSD (39.0%) being the most commonly endorsed comorbid diagnoses. A minority of participants (12.2%) received an adjustment to their psychiatric medications during the 12-week course of psychotherapy. Figure 1 displays the CONSORT diagram. Table 1 provides outcomes at each time point.

### 3.2. Primary Symptom Outcomes

#### 3.2.1. Panic

For panic on the PDSS, the omnibus test suggested a significant effect of time on panic symptoms, *F*(2, 43.98) = 19.26, *p* < 0.001). Panic symptoms decreased significantly from baseline (*M* = 17.70, *SE* = 1.14) to post-treatment (*M* = 9.96, *SE* = 1.41; *b* = 7.74, *z* = 5.49, *p* < 0.001, *d* = 1.13) and from baseline to follow-up (*M* = 10.82, *SE* = 1.38, *b* = 6.88, *z* = 4.92, *p* < 0.001, *d* = 1.00). Both effects were large. Overall, panic symptoms decreased from markedly elevated to mildly-moderately elevated, on average.

#### 3.2.2. Cognitive Anxiety

For cognitive anxiety symptoms using the STICSA-Cognitive measure, the omnibus test suggested a significant effect of time on cognitive anxiety symptoms, *F*(2, 44.01) = 7.57, *p* = 0.001). Cognitive anxiety symptoms decreased significantly from baseline (*M* = 27.57, *SE* = 1.18) to post-treatment (*M* = 23.94, *SE* = 1.39; *b* = 3.63, *z* = 2.65, *p* = 0.008, *d* = 0.51) and from baseline to follow-up (*M* = 22.33, *SE* = 1.43, *b* = 5.24, *z* = 3.69, *p* < 0.001, *d* = 0.73). Effect sizes were medium.

#### 3.2.3. Somatic Anxiety

For somatic anxiety symptoms on the STICSA-Somatic, the omnibus test suggested a significant effect of time on somatic anxiety symptoms, *F*(2, 44.66) = 7.94, *p* = 0.001. Somatic anxiety symptoms decreased significantly from baseline (*M* = 27.05, *SE* = 1.24) to post-treatment (*M* = 22.54, *SE* = 1.45; *b* = 4.51, *z* = 3.18, *p* = 0.001, *d* = 0.60) and from baseline to follow-up (*M* = 21.90, *SE* = 1.50, *b* = 5.15, *z =* 3.50, *p* < 0.001, *d* = 0.68). Effect sizes were medium.

### 3.3. Comorbid Symptom Outcomes

#### 3.3.1. PTSD

For PTSD on the PCL-5, the omnibus test suggested a significant effect of time on PTSD symptoms, *F*(2, 39.01) = 13.91, *p* < 0.001). PTSD symptoms decreased significantly from baseline (*M* = 46.36, *SE* = 3.08) to post-treatment (*M* = 32.07, *SE* = 3.67; *b* = 14.29, *z* = 4.78, *p* < 0.001, *d* = 0.77) and from baseline to follow-up (*M* = 34.97, *SE* = 3.62, *b* = 11.39, *z* = 3.89, *p* < 0.001, *d* = 0.62). Overall, comorbid PTSD symptoms decreased from moderately severe to moderate. Effect sizes were medium.

#### 3.3.2. Depression

Regarding depression outcomes on the PHQ-9, the omnibus test suggested a significant effect of time on depressive symptoms, *F*(2, 35.02) = 15.38, *p* < 0.001. Depressive symptoms decreased significantly from baseline (*M* = 16.74, *SE* = 1.00) to post-treatment (*M* = 11.67, *SE* = 1.19; *b* = 5.08, *z* = 5.35, *p* < 0.001, *d* = 0.82) and from baseline to follow-up (*M* = 13.58, *SE* = 1.20, *b* = 3.16, *z* = 3.31, *p* < 0.002, *d* = 0.51). Overall, depressive symptoms decreased from moderately severe to moderate. Effect sizes ranged from medium to large.

### 3.4. Functional Interference and Change Score Analyses

For functional inference on the IIRS, the overall omnibus test was significant, *F*(2, 41.33) = 9.24, *p* < 0.001. Functional interference decreased from baseline (*M* = 63.85, *SE* = 3.25) to post-treatment (*M* = 51.66, *SE* = 3.65, *b* = 12.19, *z* = 4.01, *p* < 0.001, *d* = 0.62) and from baseline to follow-up (*M* = 54.10, *SE* = 3.74, *b* = 9.75, *z* = 3.09, *p* = 0.002, *d* = 0.49). Overall, effect sizes were medium.

The linear regression model predicting panic change scores from baseline depression (PHQ-9) and PTSD (PCL-5) scores was not significant, *p* > 0.05.

### 3.5. Therapy Process and Satisfaction Variables

Twenty-eight participants (68.3%) completed treatment, defined as attending at least eight sessions of psychotherapy. On the PTTR, participants attended an average of 8.8 sessions (*SD* = 4.5) over an average of 12.6 weeks. Drop-out participants attended 0 sessions (*n* = 3), 1 session (*n* = 3), 3 sessions (*n* = 1), 4 sessions (*n* = 3), 5 sessions (*n* = 2), and 7 sessions (*n* = 1). The reasons for participant drop-out included: loss of contact (40%), family obligations (30%), treatment procedures—exposure (20%), and work obligations (10%). Most completing participants attended all 12 sessions (*n* = 25), with a few attending only 8 sessions (*n* = 2) or 10 sessions (*n* = 1). There were no differences in baseline symptomatology in participants that dropped out and those that completed treatment (*p*s > 0.150). Weekly average homework completion was rated at 65.3% by the treating clinicians, with an average patient understanding of 4.1 (*SD* = 1.1), patient within-session participation of 4.6 (*SD* = 1.3), and patient between-session participation of 3.8 (*SD* = 1.5). Participants rated their satisfaction with therapy at 31.7 (*SD* = 4.0) on the 35-point STTS-R therapy satisfaction scale.

## 4. Discussion

The present study investigated CBT for panic disorder via a non-controlled design in a sample of treatment-seeking veterans with panic disorder receiving care at a VAHCS. Veterans reported significant improvements for panic disorder across symptoms of panic and anxiety; comorbid symptoms of depression and PTSD; and overall impairment during CBT. Consistent with findings for civilian samples (Rayburn & Otto, 2003), primary symptom improvements were maintained for six months after psychotherapy was terminated, with medium-to-large effects, in general. In addition to clinical outcomes, the process variable findings supported the feasibility of delivering CBT for panic to veterans, with acceptable treatment completion and attendance, homework completion and engagement, and treatment engagement. For example, the treatment discontinuation rate (31.7%) was roughly consistent with CBT (27.0%), individual therapies (28.9%), and VA (27.6%) psychotherapies in veterans (Penix-Smith et al., 2025). Participants also reported high satisfaction with the intervention itself. Together, these findings provide initial support for the beneficial effects of CBT for panic disorder in veterans with panic disorder.

Consistent with the previous findings for increased symptom severity in veterans compared to community samples (Hoerster et al., 2012; Oster et al., 2017; Williamson et al., 2023), the average veteran baseline presentation of panic symptoms was consistent with being “markedly ill” on the PDSS based on studies comparing the PDSS with measures of the Clinical Global Impression Scales (Furukawa et al., 2009). In fact, baseline panic symptoms on the PDSS were roughly one standard deviation above average scores present in a similar sample of civilians (Wuyek et al., 2011). Of note, increased baseline symptomatology has been predictive of increased symptomatology at post-treatment in community samples, with authors recommending alterations to standard CBT for panic protocols to achieve better outcomes (Kampman et al., 2008). Despite these past findings in civilians and the observed symptom severity in the present findings, average treatment improvements at post-treatment and 6-month follow-up were consistent with clinical improvement between “much improved” to “very much improved” markers on the PDSS (Furukawa et al., 2009), with 60% of participants meeting criteria for at least “much improved” at post-treatment and follow-up. In addition, the observed effect sizes in veterans in the present study were consistent with reported averages for CBT for panic disorder in civilians (Gould et al., 1995). Together, these findings suggest that CBT for panic disorder was effective in veterans, potentially reducing the need for alterations to the protocol for increased symptomatology in veterans (as recommended elsewhere; Kampman et al., 2008).

In addition to elevations in symptom severity, the present study of veterans also presented with a high proportion of diagnostic comorbidity (82.9%), with two-thirds of the sample endorsing comorbid MDD. These findings for high rates of comorbidity are consistent with the general literature on veterans with mental health diagnoses (Richardson et al., 2017). Participants endorsed medium-to-large treatment effects for symptoms of depression and PTSD after CBT of panic disorder and remained present, although slightly weaker, at 6-month follow-up. These findings appear to be consistent with previous research on treating panic disorder with diagnostic comorbidity, with recommendations for remaining focused on the primary diagnosis (panic disorder), rather than shifting treatment to incorporate multiple diagnoses (Craske et al., 2007; Teng et al., 2008). However, higher rates of comorbidity found in veterans have been hypothesized as a reason for poorer treatment effects (Steenkamp et al., 2015). With the advent of newer transdiagnostic psychotherapies, greater attention has been paid to EBPs that can address primary and comorbid diagnoses simultaneously, without individualization of the treatment protocols, with data to support their use in patients with panic disorder (Gros, 2014; Barlow et al., 2017). Further study is needed to better understand the potential differences between disorder-specific protocols, disorder-specific protocols with adjustments for diagnostic comorbidity, and fully transdiagnostic protocols, especially in patient populations with high rates of comorbidity such as veterans. Of note, an initial study of transdiagnostic and disorder-specific psychotherapies on comorbid diagnoses has favored transdiagnostic protocols (Coyne & Gros, 2022).

These findings have important implications for the treatment of veterans with panic disorder. As noted previously, the prevalence rates of panic disorder are slightly higher in veteran samples compared to community samples; however, standardized assessments and psychological treatment initiatives have largely neglected panic disorder within the VA to date. Given these promising treatment findings, the incorporation of measures, such as the PDSS, in primary care and emergency care settings should be considered to improve recognition and treatment referral for panic disorder. Relatedly, approximately 25% of chest pain patients reporting in community emergency departments meet diagnostic criteria for panic disorder but are rarely recognized by emergency clinicians (Fleet et al., 1996). In addition, these promising treatment findings demonstrate that CBT for panic disorder can be useful in veterans with panic disorder, suggesting that improved dissemination and implementation efforts may be warranted within the VA (Ruzek et al., 2012).

The present study included several limitations that should be addressed in future studies of veterans with panic disorder. The sample size was insufficiently powered to more fully investigate the role of comorbidity. Given the small sample, effect sizes should be interpreted with caution. Effects may be less stable, relatively, to larger, more complete datasets using LMEMs. Results should be considered preliminary effects requiring replication in future work with larger samples of veterans with panic disorder. In addition, the study did not include a comparison group, nor randomization, in terms of assigning participants to a no treatment (e.g., wait-list control), VA treatment-as-usual for panic disorder (e.g., generalized psychotherapies), or alternative protocols for panic disorder (e.g., applied relaxation), further limiting the interpretation of the findings and introducing the potential for selection bias. Although data were collected on diagnostic reliability on the ADIS and treatment fidelity of CBT for Panic Disorder, reliability and validity data were not available for the therapist ratings on the PTTR.

Despite established efficacy in community samples, the present study extended previous preliminary studies on CBT for panic disorder in veterans via a non-controlled design by demonstrating promising results across disorder-specific symptoms (panic symptoms, anxiety sensitivity, and anxiety), comorbid presentations (symptoms of depression and PTSD), and overall functioning. The findings were present at immediate post-treatment and remained stable for 6 months after psychotherapy was terminated, although the small sample size and lack of a comparison group suggest caution regarding the interpretability of included estimates. Additional research is needed to better understand disorder-specific (CBT for Panic Disorder; Craske & Barlow, 2022) or transdiagnostic (Transdiagnostic Behavior Therapy; Gros, 2014) in veterans with panic disorder given the high rates of comorbidity observed in the population (Richardson et al., 2017).

## Figures and Tables

**Table 1:** Treatment outcomes for CBT for panic disorder.

Scale	Pre-Tx	Post-Tx	6-Month	Post-Tx *d*	6-Month *d*
Panic Measures					
PDSS-Panic	17.7 (1.1)	10.0 (1.4)	10.8 (1.4)	1.13	1.00
STICSA-Cognitive	27.6 (1.2)	23.9 (1.4)	22.3 (1.4)	0.51	0.73
STICSA-Somatic	27.1 (1.2)	22.5 (1.5)	21.9 (1.5)	0.60	0.68
Comorbid Measures					
PHQ-9-Depression	16.7 (1.0)	11.7 (1.2)	13.6 (1.2)	0.82	0.51
PCL-5-PTSD	46.4 (3.1)	32.1 (3.7)	35.0 (3.6)	0.77	0.62
Functioning					
IIRS-Impairment	63.9 (3.3)	51.7 (3.7)	54.1 (3.7)	0.62	0.49

Note. Pre-Tx, Post-Tx, and 6-Month follow-up columns are presented as means (standard errors). Tx = treatment. Comparisons were made from baseline to post-treatment and baseline to 6-month follow-up. Each symptom domain reduced significantly (*p*s < 0.05). Effect sizes are also presented in text.

## Data Availability

The data that support the findings of this study are available from the corresponding author upon reasonable request.
